# Switch to Aflibercept in Diabetic Macular Edema Patients Unresponsive to Previous Anti-VEGF Therapy

**DOI:** 10.1155/2017/5632634

**Published:** 2017-03-01

**Authors:** Filipe Mira, Manuel Paulo, Filipe Henriques, João Figueira

**Affiliations:** ^1^Centro Hospitalar Médio Tejo, Tomar, Portugal; ^2^Centro Hospitalar e Universitário de Coimbra, Coimbra, Portugal; ^3^Faculdade de Medicina da Universidade de Coimbra, Coimbra, Portugal; ^4^Associação para a Investigação Biomédica e Inovação em Luz e Imagem, Coimbra, Portugal

## Abstract

*Purpose*. The aim was to evaluate the efficacy of aflibercept in patients with diabetic macular edema (DME) unresponsive to prior anti-VEGF therapy. *Methods*. Retrospective review of DME unresponsive to previous anti-VEGF switched to aflibercept with 3 months of follow-up. Changes in best correct visual acuity (BCVA), central retinal thickness (CRT), and frequency of injections were analyzed. The percentage of subjects who had ≥20/40 (logMAR equivalent 0.3) and ≤20/200 (logMAR equivalent 1) was evaluated. *Results*. A total of 32 eyes from 26 patients were included. Mean age was 65 ± 10 years old. The mean number of previous anti-VEGF injections was 5.34 ± 2.38, and the mean number of aflibercept injections at the end of the study was 2.00 ± 0.00. The CRT at baseline was 501.47 ± 150.51 *μ*m and 367.97 ± 124.61 *μ*m at 3 months of follow-up (*P* < 0.001). The logMAR BCVA at baseline was 0.71 ± 0.36 and 0.65 ± 0.33 at the end of the follow-up (*P* = 0.037). At baseline, 12.5% of patients had ≥20/40 compared with 25% at the end of follow-up. At baseline, 28.13% of patients had 20/200 or inferior vision compared with 15.63% at the end of the follow-up. *Conclusions*. DME patients unresponsive to previous multiple ranibizumab injections demonstrate a significant anatomical and functional improvement with the switch to aflibercept.

## 1. Introduction

Diabetic macular edema (DME) is the leading cause of visual impairment in patients with diabetes mellitus and has a significant impact on quality of life [1].

Vascular endothelial growth factor (VEGF) is an important mediator of abnormal vascular permeability in DME [2, 3]. In the last few years, intravitreal injections of anti-VEGF have been established as the main treatment of DME [4–9].

Aflibercept, the latest anti-VEGF approved in ophthalmology, is composed of key domains from human immunoglobulin G1 and has approximately 100-fold greater binding affinity to VEGF-A than ranibizumab, and it also binds to VEGF-B and placental growth factor (PGF).

The DA VINCI, VISTA, and VIVID randomized clinical studies proved that aflibercept yields greater visual acuity than laser treatment [10–13]. Recently, the Protocol T, a head-to-head comparison between aflibercept, ranibizumab, and bevacizumab, showed us the superiority of aflibercept in patients with DME and poorer visual acuity [14] in the first year. In the second year, this superiority of aflibercept over ranibizumab was no longer identified [15].

Diabetic Retinopathy Clinical Research Network (DRCRnet) Protocol I showed that 52% of ranibizumab patients failed to achieve ≥2-vision-line improvement and that 40% had no resolution of retinal thickening (<250 *μ*m) at year two [16].

Studies from patients with exudative age-related macular degeneration (AMD) unresponsive to ranibizumab or bevacizumab suggested anatomical improvement with less significant visual improvement [17]. Currently, we have few data about the switch to aflibercept in DME patients unresponsive to ranibizumab or bevacizumab [18, 19].

In our department, ranibizumab is the first-line therapy for DME treatment.

Our purpose with this retrospective study was to evaluate clinical outcomes of patients unresponsive to ranibizumab that were switched to aflibercept.

## 2. Material and Methods

This retrospective review was performed at the Centro Hospitalar Médio Tejo, Ophthalmology Department, between January 2015 and January 2016.

We reviewed all patients with diagnosis of DME treated with ranibizumab (0.5 mg) that were switched to aflibercept (2.0 mg). Inclusion criteria for our study were diabetic type 2 patients aged 18 years or older with DME unresponsive to anti-VEGF with a minimum of 3 injections 4 months before switch and 3 months of follow-up. An unresponsive patient was defined as having persistent or increasing sub- or intraretinal fluid on Spectral Domain Optical Coherence Tomography (SD-OCT) after 3 or more consecutive monthly injections regardless of vision. All patients received 2 loading doses and were observed at three months.

Exclusion criteria were macular edema secondary to a cause other than diabetes, complications of diabetic retinopathy (proliferative diabetic retinopathy, tractional retinal detachment, vitreous hemorrhage, and macular ischemia), myopia greater than −6 diopters, ocular surgery 6 months prior to switch, presence of drüsens, and incomplete clinical data. SD-OCT was performed with Cirrus (Carl Zeiss Meditec, Dublin, CA, USA). The mean central foveal thickness was measured in the 1 mm central and was automatically generated. The patients and the exams were evaluated by two retina specialists, FM and MP.

Demographics, relevant clinical information, and treatment data were collected from charts. The evolution of best correct visual acuity (BCVA) and central retinal thickness (CRT) was evaluated. The proportions of the ≥20/40 and ≤20/200 patients were also assessed.

Snellen BCVA was collected and converted into logarithm of the minimal angle of resolution (logMAR).

Statistical analysis was performed using the SPSS statistical software (version 20.0 for Windows; SPSS Inc., Chicago, IL, USA). Values in the text will be represented as means ± standard deviation. A *P* value of <0.05 was considered statistically significant.

## 3. Results

A total of 32 eyes from 26 patients were included in the study. The mean age of the patients was 65 ± 10 years old, and most were females (53%). All patients were adults with type 2 diabetes. The majority (78%) was phakic, and the right eye was the prevalent eye involved (56.3%). The mean number of ranibizumab injections prior to the switch was 5.34 ± 2.38, and the mean follow-up after the switch was 3 months. The mean number of aflibercept injections after the switch was 2.00 ± 0.00 (Table 1).

**Figure 1:**
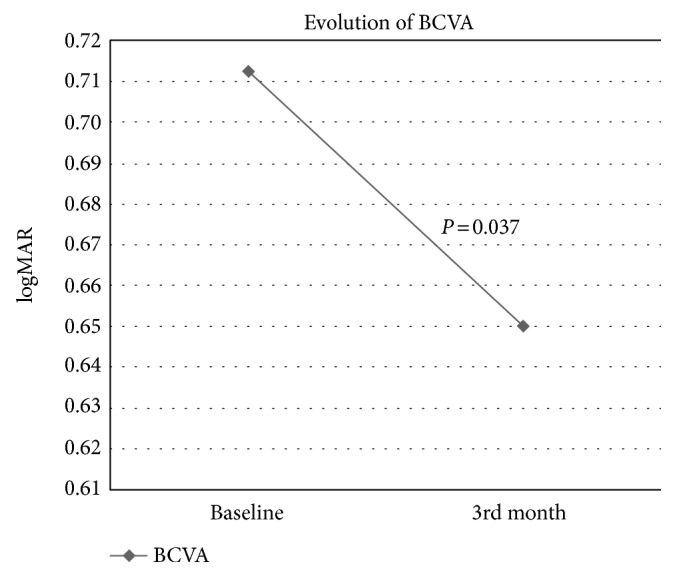
Evolution of BCVA.

The mean baseline BCVA was 0.71 ± 0.36 logMAR and was 0.65 ± 0.33 logMAR just at the end of the follow-up. As with CRT, there was a statistically significant improvement in BCVA (*P* = 0.037) (Figure 1) and there was no correlation between the BCVA at 3 months and the number of previous injections (Spearman's rho −0.135, *P* = 0.461).

**Figure 2:**
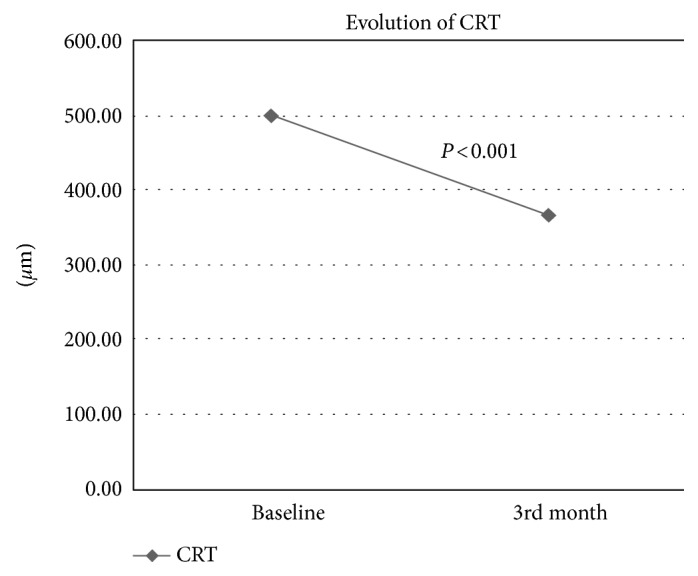
Evolution of CRT.

Regarding CRT, the mean baseline CRT was 501.47 ± 150 *μ*m and improved to 367.97 ± 124 *μ*m after 3 months. Thus, with regard to the primary endpoint, there was a statistically significant improvement in CRT (*P* < 0.001) 3 months after the switch (Figure 2). There was no correlation between the number of previous injections and CRT at 3 months (Spearman's rho −0.243, *P* = 0.180).

At baseline, 12.5% of patients had 20/40 or better vision compared with 25% at three-month follow-up.

At baseline, 28.13% of patients had 20/200 or inferior vision compared with 15.63% at the end of the follow-up.

Approximately, 63% of patients improved vision (≥1 Snellen line), 18.5% maintained vision, and 18.75% lost vision (≤1 Snellen line) at the end of the study.

During the 3-month follow-up, no ocular or systemic thromboembolic adverse events were registered.

## 4. Discussion

DME is the leading cause of visual impairment occurring in about 3 to 20% of diabetic patients [1]. Although the pathophysiology of DME is not well understood, VEGF has been identified as a major contributor [2, 20, 21].

For many years, laser treatment was considered the standard care for the treatment of these patients, contributing to a 50% reduction in vision loss. With the appearance of the VEGF blockers, the paradigm changed and now patients can gain vision.

Aflibercept was the last drug on the market, and it appears to have theoretical advantages over other drugs such as ranibizumab and bevacizumab: (1) it had a much greater binding affinity to VEGF-A; (2) it binds to growth factors, PGF 1 and 2 and VEGF-B; and (3) the vitreous half-life for aflibercept is 7.3 days, longer than that for ranibizumab (4.75 days) but slightly shorter than that for bevacizumab (8.25) [22].

We have extensive data about outcomes in patients with exudative AMD unresponsive to anti-VEGF. This data show a consistency with regard to an anatomical improvement that was not followed by a functional benefit with the switch to aflibercept [22–26].

There are limited data about the switch to aflibercept in patients with DME. Our research showed that two retrospective studies were carried out recently. Lim et al. [18] indicated a statistically significant functional and anatomical improvement in DME patients unresponsive to anti-VEGF and another undertaken by Rahimy et al. that only revealed a significant anatomical improvement [19].

Our retrospective “real world” study has some limitations: lack of control group, small sample size, short-term follow-up, and the definition of unresponsive.

Our definition of unresponsive is arbitrary but consistent with other studies: persistent or increasing sub- or intraretinal fluid on SD-OCT after 3 or more consecutive monthly injections regardless of vision [17, 27].

In our study, we demonstrate a statistically significant anatomical and functional improvement after the switch. This is relatively new in DME compared with the studies in exudative AMD [22–25]. Another interesting finding is the lack of correlation between the number of previous injections, BCVA, and CRT. Theoretically, patients with more injections have more long-standing disease and theoretically more chance to develop receptor resistance to the anti-VEGF drug used, thereafter leading to worse outcomes. The “early anti-VEGF response and long-term efficacy (EARLY analysis),” based on Protocol I, suggests that long-term response in DME patients can be assessed after 3 injections; after that, the expected improvement is minimal and switch needs to be considered [28]. Our benefit in BVCA could be explained by the early switch done in our study, at a mean of 5 previous injections, compared with 13 in the Rahimy et al. study [19].

In spite of the intrinsic limitations of this retrospective study, it gives us an overview of unresponsive DME patients in clinical practice and the benefit of switching patients to aflibercept.

## 5. Conclusion

Clinical management of an unresponsive patient to anti-VEGF is challenging for physicians. Nowadays, we do not know how to manage these patients and some questions need to be answered in the future: definition of unresponsive, time to switch, association of anti-VEGF with steroids, and, finally, the best plan of action for each patient.

The switch to aflibercept seems to be beneficial with good anatomical and functional outcome. It could also open perspectives for future randomized and prospective studies in order to clarify the best therapeutical option in these difficult cases of DME.

## Figures and Tables

**Table 1:** Demographics and clinical characteristics of patients (*N* = 32, 26 patients).

Age (years)	
Mean (SD)	65.59 (10.30)
Median (min, max)	65.50 (44, 81)
Sex	
Male	15 (46.9%)
Female	17 (53.1%)
Laterality	
Right	18 (56.2%)
Left	14 (43.8%)
Lens status	
IOL	7 (21.9%)
Phakic	25 (78.1%)
Number of preswitch anti-VEGF injections	
Mean (SD)	5.03 (2.20)
Median (min, max)	4.00 (2, 10)

SD: standard deviation; VEGF: vascular endothelial growth factor; IOL: intraocular lens.
